# Tartrate of Soda as a Purgative

**Published:** 1853-04

**Authors:** M. Delioux


From the Brit. and For. Med.-Chirurg. Rev.

*Tartrate of Soda as a Purgative.* By M. DELIOUX.

M. DELIOUX recommends the crystallized tartrate of soda as a most agreeable and certain purgative, being quite equal in power to the sulphate of soda or magnesia, and not repugnant to the taste. The medium dose, for active purgation, is ten drachms, little or no colic attending its action. The sulphate, phosphate, and tartrate of soda, and the tartrate of soda and potassa, may indeed be substituted for each other as regards their purgative action; but the tartrate of soda surpasses them all in pleasantness of taste M. Delioux is no believer in the doctrine which connects the purgative action of a body with its sapidity, the purgation resulting from the indigestibility of the body swallowed, and the exosmosis it gives rise to.

